# Impact of a Positive Viral Polymerase Chain Reaction on Outcomes of Chronic Obstructive Pulmonary Disease (COPD) Exacerbations

**DOI:** 10.3390/ijerph17218072

**Published:** 2020-11-02

**Authors:** Kulothungan Gunasekaran, Mudassar Ahmad, Sana Rehman, Bright Thilagar, Kavitha Gopalratnam, Sathish Ramalingam, Vijayakumar Paramasivam, Ashish Arora, Arul Chandran

**Affiliations:** 1 Division of Pulmonary Diseases and Critical Care, Yale-New Haven Health Bridgeport Hospital, 267 Grant Street, Bridgeport, CT 06610, USA; kavithagr@gmail.com; 2 Division of Pulmonary Diseases and Critical Care, St. Peter’s University Hospital, New Brunswick, NJ 08901, USA; mudassir738@gmail.com; 3 Department of Medicine, Shaikh Khalifa Bin Zayed Al-Nahyan Medical and Dental College, Lahore 53720, Pakistan; sana10mudassar@gmail.com; 4 Division of Hospital Medicine, Henry Ford Hospital, 2799 W Grand Blvd, Detroit, MI 48202, USA; bright.pearson@gmail.com; 5 Division of Hospital Medicine, Lovelace Medical Center, 601 Dr. Martin Luther King Jr. Ave NE, Albuquerque, NM 87102, USA; sathishmed@gmail.com; 6 Division of Nephrology, Baystate Medical Center, 759 Chestnut St, Springfield, MA 01199, USA; ronvijay@yahoo.com; 7 Division of Pulmonary Diseases and Critical Care, Saint Mary’s Hospital, 56 Franklin St, Waterbury, CT 06610, USA; docashely@yahoo.co.uk; 8 Division of Pulmonary Diseases and Critical Care, Hurley Medical Center, G-3252 Beecher Road, Flint, MI 48532, USA; arulchandranmd@gmail.com

**Keywords:** COPD, chronic obstructive pulmonary disease, respiratory virus panel, length of stay

## Abstract

Introduction: More than 15 million adults in the USA have chronic obstructive pulmonary disease. Chronic obstructive pulmonary disease (COPD) places a high burden on the healthcare system. Many hospital admissions are due to an exacerbation, which is suspected to be from a viral cause. The purpose of this analysis was to compare the outcomes of patients with a positive and negative respiratory virus panel who were admitted to the hospital with COPD exacerbations. Methods: This retrospective cohort study was conducted in the Geisinger Healthcare System. The dataset included 2729 patient encounters between 1 January 2006 and 30 November 2017. Hospital length of stay was calculated as the discrete number of calendar days a patient was in the hospital. Patient encounters with a positive and negative respiratory virus panel were compared using Pearson’s chi-square or Fisher’s exact test for categorical variables and Student’s t-test or Wilcoxon rank-sum tests for continuous variables. Results: There were 1626 patients with a total of 2729 chronic obstructive pulmonary disease exacerbation encounters. Nineteen percent of those encounters (*n* = 524) had a respiratory virus panel performed during their admission. Among these encounters, 161 (30.7%) had positive results, and 363 (69.3%) had negative results. For encounters with the respiratory virus panel, the mean age was 64.5, 59.5% were female, 98.9% were white, and the mean body mass index was 26.6. Those with a negative respiratory virus panel had a higher median white blood cell count (11.1 vs. 9.9, *p* = 0.0076). There were no other statistically significant differences in characteristics between the two groups. Respiratory virus panel positive patients had a statistically significant longer hospital length of stay. There were no significant differences with respect to being on mechanical ventilation or ventilation-free days. Conclusion: This study shows that a positive respiratory virus panel is associated with increased length of hospital stay. Early diagnosis of chronic obstructive pulmonary disease exacerbation patients with positive viral panel would help identify patients with a longer length of stay.

## 1. Introduction

Chronic obstructive pulmonary disease (COPD) is a chronic disorder characterized by the gradual progression of irreversible airflow obstruction and persistent respiratory symptoms due to airway and/or alveolar abnormalities usually caused by significant exposure to noxious particles or gases. [1]. COPD is associated with periods of sudden worsening or exacerbations that lead to emergency department visits and inpatient hospitalizations [2]. COPD exacerbations are important events in the natural course of the disease due to their adverse effects on lung function and typically increase in frequency with disease progression [3]. COPD exacerbations also pose a significant economic burden and are the leading cause of hospital admissions among COPD patients in the United States of America [4]. The total national cost attributed to COPD in 2010 was $36 billion, which accounted for direct medical expenses as well as absenteeism costs. This is projected to increase to $49 billion by the year 2020 [5]. One of the reasons for the increase in health care costs attributed to COPD is the increase in the length of hospital stay [6]. The length of hospital stay for acute COPD exacerbation tends to be between 4.5 days to 8.8 days and depends heavily on factors such as disease severity, patient characteristics, and comorbidities [7,8,9,10]. Respiratory infections are major contributors to hospitalizations for COPD exacerbations and influence their outcomes, out of which viruses are associated with approximately 50% of exacerbations [11,12]. Interestingly, symptoms from virus-related COPD exacerbations have been previously shown to last longer compared to bacteria-related exacerbations [13]. The most common viruses detected in COPD exacerbations are rhinovirus, respiratory syncytial virus, and influenza [13,14]. It has also been previously noted that patients with frequent COPD exacerbations have a greater susceptibility to viral upper respiratory infections, although the exact mechanism is unclear [11].

Although the association of COPD exacerbations with viral infections has been studied in detail, little is known about the impact of virus-associated COPD exacerbations on the length of hospital stay. The purpose of the analysis was to compare the outcomes of patients with positive and negative respiratory viral panels who were admitted to the hospital with COPD exacerbations.

## 2. Materials and Methods

The Geisinger Institutional Review Board approved the study with IRB #2018–0109. All patients between 18–80 years of age and had a history of COPD confirmed by pulmonary function test admitted with a principal diagnosis of COPD exacerbation for at least 24 h were included in this study. Patients with a history of advanced congestive heart failure (CHF), advanced malignancy, acute pulmonary embolism, using non-invasive ventilation (NIV) including continuous positive airway pressure (CPAP) for obstructive sleep apnea (OSA) at home were excluded. Since we included mechanical ventilation as one of our outcomes, we had to exclude patients who are using mechanical ventilation at baseline. According to the data broker’s information, there were initially 224,016 encounters identified with COPD diagnosis, and based on the inclusion and exclusion criteria, 2729 encounters with a COPD exacerbation were found between 1 January 2006 and 30 November 2017. Of those 2729 encounters, only 524 had a viral panel; those were included in our analysis.

Respiratory viral panel (RVP) was performed from the nasal swab, which can detect the following viruses: human metapneumovirus, influenza A (H1, H3, 2009 H1) and B, parainfluenza virus (1, 2, 3, and 4), respiratory syncytial virus, rhinovirus, adenovirus, coronavirus (HKU1, NL63, 229E, OC43, and 229E). RVP was considered as positive if any one of the viral pathogens was positive in the polymerase chain reaction (PCR). COPD exacerbations were treated with short-acting bronchodilators like ipratropium and albuterol. Providers also used systemic steroids and antibiotics, depending on the clinical scenario.

Hospital length of stay was calculated as the discrete number of calendar days a patient was in the hospital. For example, the length of stay for a patient who was admitted on 18 January and discharged on 22 January was recorded as five hospital length of stay days. ICU length of stay days was calculated using a similar method. Because few patients had mechanical ventilation (MV) during their hospitalization, it was described as a binary categorical variable. It was also described as the number of days a patient had MV and as the proportion of hospital stay days free of MV.

### Statistical Analysis Plan

Categorical variables are described using frequency counts, and percentages and continuous variables are described using means and standard deviations (S.D.) or medians and interquartile ranges (IQR). Descriptive statistics are provided for all 524 encounters with respiratory viral panels performed. Patient encounters with positive and negative RVPs were compared using Pearson’s chi-square or Fisher’s exact test for categorical variables and Student’s t-test or Wilcoxon rank-sum tests for continuous variables. The association between RVP results and intensive care unit (ICU) admission, mechanical ventilation, and inpatient mortality is described using frequency counts, and percentages and the association tests were done with Pearson’s chi-square and Fisher’s exact tests, as appropriate. The length of ICU stays for encounters involving an ICU stay was compared using Student’s t-test. Hospital length of stay and mechanical ventilation days for those on mechanical ventilation were compared using Wilcoxon rank-sum tests because of the outcome variables’ skewed distributions. Poisson regression models were used to estimate the predicted length of stay with 95% confidence intervals for RVP status, sex, 25th, 50th, and 75% percentiles of age. A multivariable Poisson regression model was used to adjust for age and sex. Logistic regression models were used to estimate the odds ratios and 95% confidence intervals for RVP status, age, sex, and respiratory rate. Results from multivariable logistic regression models adjusted for age and sex..

## 3. Results

There were 1626 patients with a total of 2729 COPD exacerbation admissions. Nineteen percent of those encounters (*n* = 524) had a respiratory viral panel performed during their admission. Among these encounters, 161 (30.7%) had positive results, and 363 (69.3%) had negative results. For encounters with RVPs, the mean age was 64.5, 59.5% were female, 98.9% were white, and the mean body mass index (BMI) was 26.6. Patient encounters with positive and negative results were significantly different with respect to their white blood cells (WBC) values. Those with negative RVPs had a higher median WBC (11.1 vs. 9.9, *p* = 0.0076). There were no other statistically significant differences in characteristics between the two groups (Table 1).

Positive RVP patients had a significantly more extended hospital stay. There were no significant differences with respect to being on mechanical ventilation or mechanical ventilation-free days. Patients were only on mechanical ventilation for 6.7% of encounters. Five encounters (1.0%) resulted in an inpatient death during the hospital stay. Three patients with positive RVP and two with negative RVPs died. One additional positive RVP patient died within 30 days of their hospital discharge (Table 2).

Mean hospital length of stay days were estimated with Poisson regression and showed that patient encounters with positive RVPs had higher stay days than those with negative results (mean stay days of 5.63 vs. 4.93, *p* = 0.0051). After adjusting for age and sex, the difference persisted. The mean hospital length of stay days was 5.69 days for positive RVP patients and 4.94 days for negative RVP patients (*p* = 0.0032) (Table 3).

Of 524 patients, 77 patients had a sputum culture based on the providers’ clinical judgment at that time. There was no statistical difference in the length of the stay between positive and negative sputum cultures in those patients with a sputum culture using the Spearman’s rho correlation coefficient (Table 4).

## 4. Discussion

This is a retrospective study evaluating the relationship between the detection of viral infections in COPD exacerbations and comparing the characteristics of positive viral exacerbations as opposed to negative viral exacerbations.

In our study, of the 19% of patients with COPD exacerbation tested, 30.7% of those had positive RVP, similar to most other studies that used RT-PCR to detect viral infection in patients with COPD exacerbation [13,15,16,17]. Similar to other studies, the burden of viral diseases on COPD exacerbations is an underestimated value. This could probably be explained by the different rates of RT-PCR performance for different viruses, ordering RT-PCR mostly during the influenza season, and shorter period of virus shedding in adults, resulting in early negative seroconversion. Even though RT-PCR has been shown to have excellent sensitivity and specificity, De Serres et al. [15] reported that a low rate of rhinovirus infection in his study might be due to lower RT-PCR sensitivety, epidemiological season, and selected population like hospitalized patients. Nonetheless, we have ample understanding that clinically, upper respiratory tract symptoms precede a true exacerbation. Some studies have shown that at least one virus detection by RT-PCR in 64% of patients admitted with acute exacerbation of COPD and noted that these patients had a higher frequency of exacerbation than in patients without a detectable virus. [13] Regarding the type of virus detected, Dai et al. showed that influenza is the commonest viral infection detected in acute exacerbation of COPD [18]. Later, Zwaan’s et al. showed that rhinoviruses (16%) that are less tested for are often more commonly detected than RSV (9%) and influenza (7%) in an analysis of 1728 patients from 19 studies [19]. Unfortunately, we did not have patient-level data, so we could not conclude which viral infections were prevalent in our patient population.

In our study, patients who had a positive RVP had a longer length of hospital stay, which is similar to findings noted in a study demonstrating that the presence of higher levels of sputum inflammatory markers in the setting of viral infection results in much more severe exacerbations [20]. Hewitt et al. showed that the natural antiviral response is diminished in airway disease, and this often leads to longer exacerbation episodes [21]. This could probably be due to a change in the Th1/Th2 ratio and a lower IL-17 and IFN- γ, causing persistent symptoms in our patients with a positive RVP and an area that requires further study [22,23,24,25]. Similarly, Seenmungal et al. had shown that the increased length of hospitalization with a positive RVP could be due to the longer duration taken for symptom resolution [26]. The incidence and burden of increased symptoms due to a virus-associated exacerbation are greater than exacerbation without viral infection, leading to a longer hospital stay.

About 25% of hospitalized patients with an exacerbation have been noted to have a co-existing bacterial infection [14]. Still, in our patient group, even though there is a difference in the length of stay between the positive and negative bacterial sputum culture, it is not statistically significant. Interestingly, only 77 of our patients had sputum culture; this might be because of the fact the baseline procalcitonin was low in our patient, which might have precluded the physicians from ordering sputum culture.

Another important finding in our study was that only 19% of the total acute exacerbation of COPD admissions had an RVP performed. The routine testing for viruses in patients presenting with COPD exacerbation is not common clinical practice due to the lack of upper respiratory tract symptoms, clinicians’ unawareness about the role of viral infections, and utilization of this testing mostly during influenza season. These factors might have led to low testing of viral causes of COPD exacerbation in our patient population. On the contrary, there is a huge amount of accumulating evidence over the past few years, demonstrating the impact of viral infections on COPD exacerbations. As we gain a more in-depth understanding of the pathophysiology and mechanisms that drive the process, it has become more imperative to develop clinical protocols to include early detection of these infections. In addition, patients with concomitant viral and bacterial infection (rhinovirus and hemophilus influenza) were noted to have a greater decline in forced expiratory volume in one second (FEV1) and an increase in inflammatory markers, suggestive of more severe infection [27]. This highlights that even in patients with COPD exacerbation from a proven bacterial infection, it is imperative to check a viral panel to predict the lung function decline [28]. Further, in the same way, a positive RVP combined with negative markers for bacterial infection may also reduce the inadvertent use of antibiotics in patients with COPD exacerbation and thereby reduce the incidence of antibiotic resistance patterns.

It is clear from this study that the true burden of viral infections on acute exacerbation of COPD is underestimated if only 19% of the cases were tested. This highlights the importance of more extensive testing to detect viral infections, which would have a huge impact on treatment and medical decision making. Implementing steps to prevent the spread of viral infections is also an important consideration, especially given that viruses play an important role in disease progression.

## 5. Limitations

There are some limitations to our study. First, this is a retrospective cohort study and limited to data available on hand. Even though we excluded patients with specific comorbidities that can affect the length of stay, there could still be some underlying comorbidities that might have had a confounding effect. We also do not have patient-level data on the eosinophils count, type of viruses, or specific treatment received, which may have impacted the length of stay. Although we had smoking status information regarding patients, we do not have data on oxygen supplementation. Our cohort also included predominantly a white population, which is different from other studies looking at COPD length of stay [29]. Our data on social determinants of health were limited as well.

## 6. Conclusions

Our study shows that a positive respiratory virus panel is associated with increased length of hospital stay in this specific group at the Geisinger Medical Center. This study also emphasizes the need for a viral polymerase chain reaction testing on all the chronic obstructive pulmonary disease (COPD) exacerbation patients, which would enable us to predict the group of patients who would require a higher length of stay.

## Figures and Tables

**Table 1:** Summary of characteristics for all encounters.

Clinical Characteristics	Encounters Overall		Positive RVP		Negative RVP		*p*-Value
	(*n* = 524)		(*n* = 161)		(*n* = 363)		
	*n*	%	*n*	%	*n*	%	
Patients, *n*	524		161(30.7%)		363(69.3%)		
Admission Age, Mean (S.D.)	64.5 (9.4)		63.9 (10.4)		64.7 (8.9)		0.4217
BMI, Mean (S.D.)	26.6 (7.3)		27.2 (8.1)		26.3 (6.9)		0.2427
Sex							0.4248
Female	312	59.50%	100	62.10%	212	58.40%	
Male	212	40.50%	61	37.90%	151	41.60%	
Race							0.1843
Others	6	1.10%	0	0.00%	6	1.70%	
Caucasian	518	98.90%	161	100.00%	357	98.30%	
Smoking Status							0.1344
Current	236	45.00%	82	50.90%	154	42.40%	
Former	259	49.40%	69	42.90%	190	52.30%	
Never	29	5.50%	10	6.20%	19	5.20%	
Insurance Type							0.5547
Geisinger Health Plan	217	41.40%	64	39.80%	153	42.10%	
Medicaid/Medicare/Veterans	226	43.10%	68	42.20%	158	43.50%	
Commercial	81	15.50%	29	18.00%	52	14.30%	
Encounter Type							0.4061
ED to Inpatients	502	95.80%	156	96.90%	346	95.30%	
Inpatients Only	22	4.20%	5	3.10%	17	4.70%	
Discharge Disposition							0.1071
Home/Home with Health	444	84.70%	130	80.70%	314	86.50%	
Other (Rehab Center/Long Term Facility)	75	14.30%	28	17.40%	47	12.90%	
Inpatient Death	5	1.00%	3	1.90%	2	0.60%	
Positive Bacterial Sputum Culture							1.0000
Yes	75	97.4%	20	100.0%	55	96.5%	
No	2	2.6%	0	0.0%	2	3.5%	
Vitals and labs							
Systolic BP, Mean (S.D.)	140.3 (24.8)		139.6 (22.3)		140.6 (25.8)		0.6439
Heart Rate, Mean (S.D.)	103.2 (20.2)		103.5 (18.8)		103.1 (20.8)		0.8459
WBC, Median (IQR)	10.7 (8.1,14.4)		9.9 (7.2,14.2)		11.1 (8.5,14.6)		0.0076
Pro BNP	281 (113, 720)		265 (120, 592)		299 (113, 811)		0.6538
D-Dimer	0.500 (0.325, 1.145)		0.510 (0.320, 1.100)		0.490 (0.330, 1.190		0.8413
Procalcitonin, median (IQR)	0.07 (0.05, 0.16)		0.07 (0.05, 0.13)		0.08 (0.05, 0.17)		0.2588
Troponin, median (IQR)	0.01 (0.01, 0.03)		0.01 (0.01, 0.03)		0.01 (0.01, 0.03)		0.7219

RVP = respiratory viral panel; S.D = standard deviations; BMI = body mass index; ED = emergency department; systolic BP = systolic blood pressure; WBC = white blood cells; IQR = interquartile ranges; pro BNP = pro brain natriuretic peptide.

**Table 2:** Outcomes for all encounters by the positive and negative viral panel.

Outcomes	Encounters Overall (*n* = 524)		Positive RVP (*n* = 161)		Negative RVP (*n* = 363)		*p*-Value
	*n*	%	*n*	%	*n*	%	
Hospital LOS Days, Median (IQR)	4 (3, 6)		5 (3, 7)		4 (3, 5)		0.0421
ICU Stay							0.5564
Yes	28	5.30%	10	6.20%	18	5.00%	
No	496	94.70%	151	93.80%	345	95.00%	
ICU LOS Days, Mean (S.D.)	4.8 (1.9)		5.5 (1.8)		4.4 (1.9)		0.1732
Mechanical ventilation							0.6365
Yes	35	6.70%	12	7.50%	23	6.30%	
No	489	93.30%	149	92.50%	340	93.70%	
Mechanical Ventilation Days, Median (IQR)	3 (2, 4)		4.5 (2, 5)		3 (2, 3)		0.1171
Inpatient Death							0.1718
Yes	5	1.00%	3	1.90%	2	0.60%	
No	519	99.00%	158	98.10%	361	99.40%	

RVP = respiratory virus panel; IQR = interquartile ranges; ICU = intensive care unit; LOS = length of stay; S.D = standard deviation.

**Table 3:** Hospital length of stay day’s crude and adjusted multivariable model results.

Variables	Hospital Length of Stay Days Unadjusted			Hospital Length of Stay Days Adjusted for Age and Sex		
	Mean Estimate LOS Days	95% CI for Mean	*p*-Value	Mean Estimate LOS Days	95% CI for Mean	*p*-Value
RVP			0.0051			0.0032
Positive	5.63	(5.22, 6.08)		5.69	(5.27, 6.14)	
Negative	4.93	(4.66, 5.20)		4.94	(4.68, 5.22)	
Age			0.0201			0.0158
Age = 58 (Q1)	4.95	(4.68, 5.23)		5.10	(4.82, 5.40)	
Age = 64 (Median)	5.12	(4.90, 5.36)		5.28	(5.04, 5.54)	
Age = 72 (Q3)	5.36	(5.07, 5.67)		5.54	(5.22, 5.87)	
Sex			0.1387			0.1196
Female	5.00	(4.71, 5.30)		5.11	(4.82, 5.43)	
Male	5.35	(5.00, 5.74)		5.49	(5.12, 5.89)	

CI = confidence interval; LOS = length of stay; RVP = respiratory virus panel; Q = quartile.

**Table 4:** Hospital length of stay for positive sputum culture.

Positive Bacterial Sputum Culture	*n*	Median Hospital LOS Days	*p*-Value: 0.4458
Yes	75	5 (3, 7)	
No	2	6 (5, 7)	

## Abbreviations

COPD	Chronic obstructive pulmonary disease
RVP	Respiratory virus panel
PCR	Polymerase chain reaction
IFN	Interferon
IL	Interleukin
Th	T helper cells
